# Chorangiosis placenta with 5-vessel umbilical cord with omphalomesenteric duct remnant: An unusual association

**DOI:** 10.4274/tjod.37531

**Published:** 2019-01-09

**Authors:** Neha Garg, Preeti Diwaker, Shubhra Aggarwal, Jyotsana Harit Gaur

**Affiliations:** 1 University College of Medical Sciences, Guru Teg Bahadur Hospital, Clinic of Pathology, New Delhi, India

**Keywords:** Placenta, umbilical cord, chorangiosis

## Abstract

Placenta is an organ that is responsible for nourishing and protecting the fetus during pregnancy. Histologic examination of the placenta can yield significant information about pre-uterine and uterine conditions affecting fetal growth. Chorangiosis is defined as the presence of ≥10 terminal villi, each containing ≥10 capillaries per terminal villus in ≥10 low power (×10) fields in at least 3 or more random, non-infarcted cotyledons of the placenta. Chorangiosis is an adaptive response to in-utero hypoxia and its presence signifies better pregnancy outcomes. Abnormalities that lead to multiple cord vessels are rare with the majority of reported cases highlighting four vessels due to a persistent right umbilical vein. We report here a case of chorangiosis of placenta associated with a rare 5-vessel umbilical cord and omphalomesenteric duct remnant. To the best of our knowledge, this is the first case report to document such an association.

**PRECIS:** We report here a case of chorangiosis of placenta associated with the rare 5-vessel umbilical cord and omphalomesenteric duct remnant.

## Introduction

The placenta is an organ that is responsible for nourishing and protecting the fetus during pregnancy. Histologic examination of placenta can yield significant information about pre-uterine and uterine conditions affecting fetal growth. One of the histo-morphologic features to be evaluated is the vascularity of chorionic villi. The number of vascular channels present in terminal villi ranges from 2 to 6 and if the number increases beyond this then it is termed as ‘villous hypervascularity^(1)^.’ The term ‘chorangiosis’ was introduced by Altshuler in 1984 and is defined as the presence of ≥10 terminal villi, each containing ≥10 capillaries per terminal villus in ≥10 low power (10´) fields in at least 3 or more random, non-infarcted cotyledons of the placenta^(2)^. Chorangiosis is known to occur as an adaptive response to chronic low-grade placental hypoxia^(1)^. It has been found to be associated with various maternal, fetal and placental disorders^(1)^. Rarely, its association has also been demonstrated with umbilical cord anomalies such as true and false knots, long cord, umbilical vein dilatation or thrombosis, nuchal cord, and single umbilical artery^(3)^. Herein, we report a case of chorangiosis of placenta associated with the rare 5-vessel umbilical cord.

## Case Report

**Figure 1:**
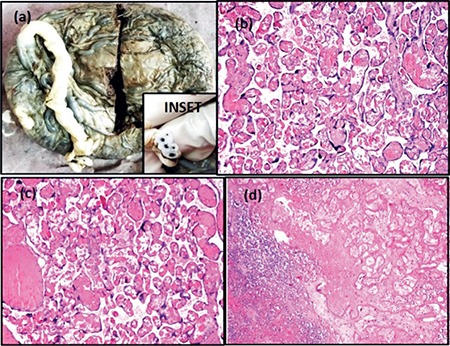
(a) Gross appearance of the placenta with attached umbilical cord. Inset showing umbilical cord with 5 blood vessels, (b, c) (100x) H/E-stained sections from the placenta showing ≥10 capillaries each in ≥10 terminal villi in ≥10 non-infarcted areas, (d) (100x) Sections from hemorrhagic areas showing ischemic necrosis

**Figure 2:**
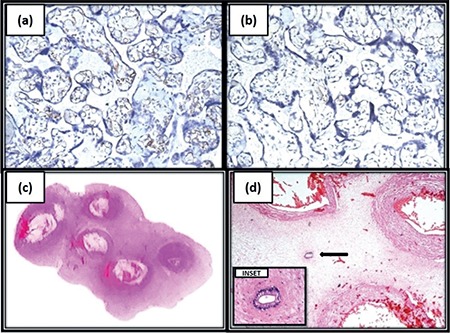
(a) (200x) Immunohistochemically stained capillary endothelial cells showed uniform positivity with CD34, demonstrating more capillaries than were easily discernible by hematoxylin-eosin stain, (b) (200x) Stain for smooth muscle actin was negative, (c) Sections from umbilical cord showing 5 blood vessels; 4 arteries and 1 vein and (d) (40x) an omphalomesenteric duct remnant is seen in between the blood vessels. INSET (400x) shows omphalomesenteric duct remnant

A 27-year-old female, para-3-live-1, with gestational hypertension and oligohydramnios presented to the gynecologic emergency at 34 weeks’ gestation in labor. Emergency lower segment cesarean section was perfomed in view of fetal distress. The child was born with a birth weight of 1.6 kg and was stable. There were no congenital anomalies in the child. The placenta was sent for histopathologic examination. On gross examination, the placenta was complete and measured 12´11´3 cm with the attached umbilical cord measuring 18 cm in length. The umbilical cord contained 5 blood vessels (Figure 1a). On microscopy, sections from the placenta revealed ≥10 capillaries each in ≥10 terminal villi in ≥10 non-infarcted areas examined in ≥3 low power (10´) fields of placenta (Figures 1b, c). Immunohistochemically, the capillary endothelial cells showed uniform positivity with CD34, demonstrating more capillaries than were easily discernible using hematoxylin-eosin staining (Figure 2a), and staining for smooth muscle actin (SMA) was negative (Figure 2b). There was no evidence of increased cellularity or fibrosis in the stroma. Sections from hemorrhagic areas showed ischemic necrosis (Figure 1d). Sections from the umbilical cord showed 5 blood vessels; 4 arteries and 1 vein (Figure 2c) and an omphalomesenteric duct remnant (Figure 2d). Placental membranes were histopathologically unremarkable. A diagnosis of chorangiosis placenta with 5 blood vessels and omphalomesenteric duct remnant in the umbilical cord was given.

## Discussion

Chorangiosis is a very rare entity found in 5-6% of placentas^(2)^. Its incidence increases with gestational age with more cases found in late preterm (32 to 37 weeks) and term (>37 weeks) pregnancies^(1,2)^. Its presence has been correlated with fetal morbidity and mortality and congenital malformations as high as 42% and 39%, respectively^(1)^.

The proposed pathogenetic mechanism of chorangiosis is that chronic hypoperfusion or tissue hypoxemia causes elaboration of vascular endothelial growth factor, platelet-derived growth factor, and transforming growth factor-b by mesenchymal and trophoblastic cells^(3)^. An alternative hypothesis highlighting the role of macrophage-derived tumor necrosis factor-a has also been suggested^(3)^. Increased intramural pressure due to umbilical vein obstruction is also thought to play a role in the development of chorangiosis in cases associated with cord anomalies, such as long umbilical cord and thrombosis of vessels^(3)^.

Chorangiosis is a histopathologic diagnosis. It must be differentiated from placental congestion, tissue ischemia, chorangioma and chorangiomatosis^(3,4)^. In placental congestion, the vasculature is numerically normal. In tissue ischemia, there is shrinkage of the villi. Chorangioma is a well-circumscribed mass of solitary or multiple nodules. On microscopy, it is comprised of capillary-sized vascular channels with a mixture of endothelial cells, stromal cells, and surrounding trophoblasts. Chorangiomatosis is a heterogeneous, less well-defined lesion with intermediate features between chorangioma and chorangiosis. It has hyperplastic capillaries surrounding larger vessels in the central core of stem villi with increased numbers of loose, poorly cohesive lattices of perivascular bundles of reticulin fibers and circumferential layers of pericytes. These pericytes stain positively for SMA. Also, both chorangioma and chorangiomatosis are seen before 32 weeks of gestation and involve more proximal elements of villous structures, whereas chorangiosis is more common in late preterm and term pregnancy, and is a diffuse process involving the terminal villi.

Its etiology is poorly understood, but it has been found to be associated with various maternal and fetal disorders^(1)^. Placental disorders such as placentomegaly, chronic villitis, acute chorioamnionitis, amnion nodosum, and placenta previa have also been reported in association with chorangiosis^(1,3)^. However, contrary to Altshuler’s initial description, it is now suggested to be associated with much improved pregnancy outcomes. Recently in 2016, Stanek reported that chorangiosis is an adaptive and protective mechanism against in utero hypoxia, and abnormal pregnancy outcomes are not a consequence of chorangiosis per se^(1)^. In 2017, Petersen et al.^(5)^ concluded that chorangiosis is a placental marker of antepartum chronic low-grade hypoxia. In the present case study, the patient had gestational hypertension, oligohydramnios, and delivered a preterm stable child. The indication for cesarean section was decreased fetal heart rate with fetal distress, which might have been be due to chronic hypoxia resulting from pre-eclampsia.

Also, in the present case, an association of chorangiosis with a multiple vessel umbilical cord, having 5 blood vessels with an omphalomesenteric duct remnant was identified. Abnormalities that lead to multiple vessels in the cord are rare with the majority of the reported cases highlighting four vessels due to a persistent right umbilical vein^(6)^. However, occasional case reports mention five or more vessels in the cord in association with conjoined twins^(6)^. The child in the present case had no congenital anomalies. Also, the literature regarding their possible mechanism of occurrence and significance is sparse.

The omphalomesenteric (vitelline) duct connects the midgut lumen with the yolk sac in the developing fetus. It is often associated with remnants of vitelline vessels, seen in about 7% of umbilical cords^(7)^. Microscopically, they are lined by cuboidal to columnar epithelium with an intestinal phenotype and may have a surrounding smooth muscle layer.

To conclude, chorangiosis is an important histopathologic sign of fetal injury. It is an adaptive response to in-utero hypoxia and its presence signifies better pregnancy outcomes. To the best of our knowledge, this is the first case report to document the association of chorangiosis with a 5-vessel umbilical cord, whose presence does not always herald an adverse perinatal outcome, and an omphalomesenteric duct remnant.
